# Natural History of Man

**Published:** 1842-04

**Authors:** 


Art. X.—*Natural History of Man.* By J. C. Prichard, m.d. f.r.s. m.r.i.a. &c. &c. Parts I-II-III.—*London,* 1842. 8vo, pp. 160.

All our readers must be familiar with the great classical work of Dr. Prichard, on the Physical History of Mankind, which we have had occasion, more than once, to notice in our pages, with unmixed praise. That work is for men of science, and has been received by them as the highest authority on the subjects of which it treats. A work of a somewhat similar kind, but fitted for the general reader, was still wanting in the English language; and it is to supply this defect that the present treatise has been composed. And " in order to render it acceptable and useful to general readers, the author has omitted all anatomical and physiological details that were not absolutely requisite for the setting forth of his principal argument; and such facts of this kind as were indispensable he has stated in the most brief and general manner. The whole work will be comprised in one volume octavo, and will be completed in ten monthly parts. It will contain an account of all the races of men which are distinguished by any remarkable peculiarities; and these peculiarities will be illustrated by a large collection of engravings, taken partly from a series of original portraits and in part selected from the late splendid publications of French, German, Dutch, and Russian voyagers." As far as the work has gone it has entirely fulfilled the promise of the prospectus. We can give it no higher praise than to say, that it is worthy of its author and of his preceding works. We earnestly recommend it to all our readers. The parts hitherto published are beautifully and copiously illustrated with woodcuts and coloured plates.

On one point we must remonstrate—the *very high price* of the work: and here we expect the publisher and not the author must bear the blame. The work is certainly handsomely got up, and the expense of the plates must be very considerable, but to pay half-a-crown for 48 pages, each not containing more than one half of our own, is much beyond the mark. At the same rate, this Journal, instead of *six*, would cost *thirty* shillings! We think the sale will be greatly circumscribed by this ill-judged dearness.

